# Detection of ALV p27 in cloacal swabs and virus isolation medium by sELISA

**DOI:** 10.1186/s12917-019-2150-z

**Published:** 2019-10-30

**Authors:** Xiaoyu Zhou, Lin Wang, Anning Shen, Xi Shen, Moru Xu, Kun Qian, Hongxia Shao, Yongxiu Yao, Venugopal Nair, Jianqiang Ye, Aijian Qin

**Affiliations:** 1 Ministry of Education Key Lab for Avian Preventive Medicine, Yangzhou University, No. 12 East Wenhui Road, Yangzhou, Jiangsu 225009 People’s Republic of China; 2 Jiangsu Co-innovation Center for Prevention and Control of Important Animal Infectious Diseases and Zoonoses, No. 12 East Wenhui Road, Yangzhou, Jiangsu 225009 People’s Republic of China; 3 Jiangsu Key Lab of Zoonosis, No. 12 East Wenhui Road, Yangzhou, Jiangsu 225009 People’s Republic of China; 4 The Pirbright Institute & UK-China Centre of Excellence on Avian Disease Research, Ash Road, Pirbright, Surrey GU24 0NF UK

**Keywords:** Avian leukosis virus, Novel subgroup K, Enzyme-linked immunosorbent assay, Meconium

## Abstract

**Background:**

Avian leukosis (AL), which is caused by avian leukosis virus (ALV), has led to substantial economic losses in the poultry industry. The kit used to detect all ALV-positive chickens in breeder flocks is very important for efficiently controlling AL. However, a new emerging ALV subtype is currently a severe challenge in the poultry industry.

**Results:**

In this paper, we compared different enzyme-linked immunosorbent assay (ELISA) kits for detecting p27 of ALV in the same batch of meconium samples. Different positive samples were further analyzed by PCR or virus isolation. The results showed that 36 positive samples among the 1812 chicken meconium samples could be detected by a sandwich ELISA (sELISA) kit, but only 17 positive samples could be identified by a commercial kit. To verify this result, cloacal swabs and viruses isolated from the positive chickens (2 days old) were used to detect the presence of p27. The results showed that the positive rate of p27 was 100% for the swabs and 40% for virus isolation. Surprisingly, PCR and sequence analysis revealed that the *env* gene of ALV in these positive samples belonged to the novel subgroup K (ALV-K).

**Conclusion:**

These data not only demonstrate the relatively high sensitivity of the sELISA kit but also highlight the challenge of controlling ALV-K.

## Background

Avian leukosis virus (ALV), which belongs to the family *Retroviridae*, subfamily *Orthoretrovirinae*, and genus *Alpharetrovirus*, is known to cause significant economic losses in the poultry industry worldwide due to virus-associated neoplasia and reduced productivity in commercial layer and breeder flocks [1]. Therefore, the eradication of ALV is crucial in the poultry industry. ALV eradication was realized in developed countries three decades ago; however, ALV subgroup J (ALV-J) still emerged and was first isolated in 1989 [2]. To date, ALV-J has spread around the world, leading to epidemics in many countries, including China. In 1991, ALV-J was identified in the developed countries where ALV was successfully eradicated with a commercial enzyme-linked immunosorbent assay (ELISA) kit [3]. Decades later, ALV subgroup K (ALV-K) emerged in China, where commercial ELISA kits have been extensively used for ALV detection. Although commercial ELISA kits have been suggested to be effective, there is a potential link between the emergence of new ALV subgroups and the inability to detect ALV in the meconium by commercial ELISA kits.

Recently, ALV-K was isolated and identified in “yellow meat-type chickens” in China [4]. How can ALV be eradicated in domestic breeder chicken flocks? The key principle is to detect and remove all infected chickens, particularly by evaluating the meconium in 1-day-old chickens, to avoid early horizontal spread among young chickens [3]. However, we found that false negative results for ALV frequently occurred with some ELISA kits that are commonly used in ALV eradication programs when we compared the effectiveness of different kits. Although many molecular biology and immunology methods for ALV detection have been reported in recent years [5–8], in this study, we compared a sandwich ELISA (sELISA) kit based on monoclonal antibodies (mAbs) specific for the capsid protein p27 of ALV [5] with a commercial ELISA kit by analyzing the meconium of one-day-old chickens. The results showed that the sELISA kit was more sensitive than the commercial ELISA kit, which indicates the importance of developing a more sensitive ELISA kit for the detection of emerging novel subgroups of ALV.

## Results

### sELISA kit could more efficiently detect ALV-positive samples than a commercial ELISA kit

In this study, we compared an sELISA kit to a commercial ELISA kit by simultaneously detecting p27 of ALV in the same batch of 1812 meconium samples. Only 17 positive samples were detected by the commercial kit, but 36 of the samples, including all 17 positive samples found using the commercial kit, were found to be positive by the sELISA kit.

### Confirmation of the positivity of samples detected by the sELISA but not by the commercial ELISA

**Fig. 1:**
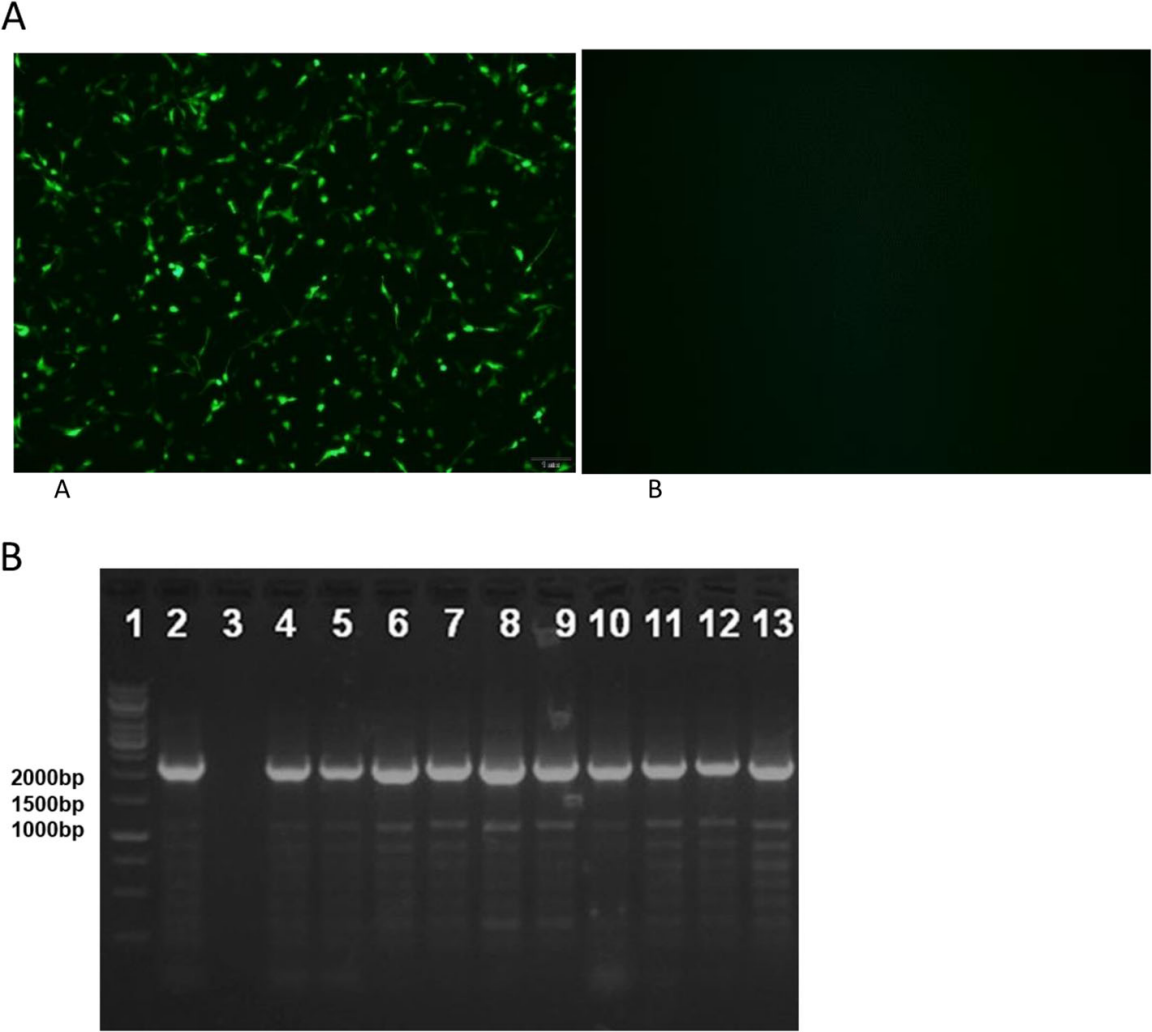
Results for different samples evaluated by an IFA and PCR. **a** IFA for identifying DF-1 cells infected with viral isolates using the monoclonal antibody 5D3; A, DF-1 cells infected with an isolate; B, noninfected DF-1 cells. **b** PCR results for the *env* genes of the isolates. Lane 1: 1-kb DNA marker; Lane 2: positive control; Lane 3: negative control; Lane 4–13: amplified *env* genes from samples 1–10, respectively

To confirm the high sensitivity and reliability of the sELISA, 10 chickens with meconium samples that were positive by the sELISA kit but negative by the commercial ELISA kit were selected for further detection. Cloacal swabs and peripheral blood and spleen samples were collected from these ten chickens. Detection of the p27 antigen by sELISA showed that all ten cloacal swabs were positive. Viral isolation showed that 4 of the 10 peripheral blood samples were positive (Table 1). The four isolated ALV strains were also confirmed by an immunofluorescence assay (IFA). As described in Fig. 1a, DF-1 cells infected with these isolates specifically reacted with the mAb 5D3 specific for p27 of ALV, as shown by bright green fluorescence. All these data clearly demonstrated that the positive samples identified by sELISA contained ALV.

**Table 1:** ELISA results for ALV p27 in cloacal swabs evaluated by the sELISA kit and virus isolation ^a^ S/P, OD_650_ values of a sample/OD_650_ value of the positive control

Sample number	Cotton swab		Positive rate	Isolation of virus (tissue or blood)		Positive rate
	S/P	Result		S/P	Result	
1	0.198	+	100%	0.063		40%
2	0.195	+		0.059		
3	0.469	+		0.057		
4	0.203	+		0.197	+	
5	0.218	+		0.229	+	
6	0.221	+		0.098		
7	0.433	+		0.201	+	
8	0.231	+		0.072		
9	0.410	+		0.069		
10	0.277	+		0.318	+

### Novel ALV subgroup was found in the samples positive by sELISA but negative by commercial ELISA

**Fig. 2:**
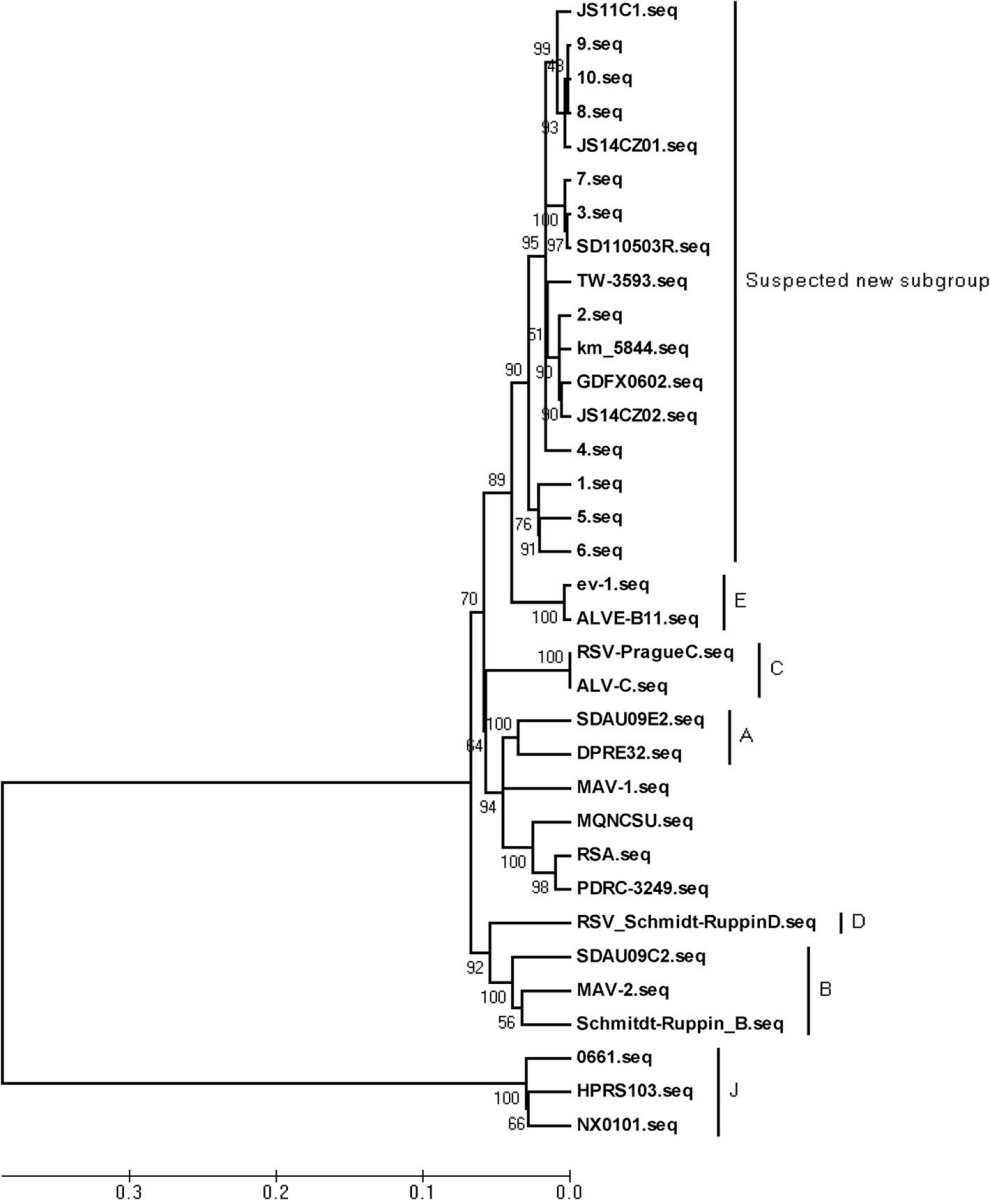
Comparison of e*nv* gene sequences of the positive samples (1–10) with those of other ALVs. Phylogenetic tree analysis using the neighbor-joining method (bootstrap method with 1000 replicates). ALV subgroups A-E and J are shown on the right. Bars, substitutions per nucleotide position

To further identify the ALV subgroups in the samples identified as positive by sELISA but negative by commercial ELISA, genomic DNA was extracted from tissue samples (liver or spleen mixtures) (samples 1, 2, 3, 6, 8 and 9) or DF-1 cells infected with the four ALV isolates. A 2200-bp fragment covering the *env* gene of ALV was amplified by PCR using the extracted DNA as a template as described in Fig. 1b. Sequence analysis of the *env* gene showed that the isolates were phylogenetically close to the novel subgroup ALV strains JS11C1 and JS14ZC02 (Fig. 2). All these data demonstrated that the ALV viruses identified in all 10 samples belonged to the novel K subgroup of ALV.

## Discussion

It is well known that ELISA kits for p27 antigen detection have played an important role in ALV eradication in recent years. Many different kits for p27 antigen detection have been developed and are widely used worldwide. Since 2000, ALV-J has been the dominant ALV subtype in China. Due to the effective eradication program for ALV, only a few ALV-J cases have been found on some farms in China [9]. However, the emergence of ALV-K creates challenges for ALV detection, and an eradication program has recently begun [2, 10, 11]. In 2012, Wang et al. isolated three novel ALV strains named JS11C1, JS11C2 and JS11C3 from an indigenous Chinese flock of LuHua chickens [4]. The gp85 sequences of the three ALV strains were different from those of strains in the other subgroups, hence, these strains have been proposed to comprise a new subgroup, ALV-K. According to the detection of different subgroups of ALV, we found that the sELISA kit was more sensitive than other kits in detecting ALV-J and ALV-A (see Table 2 for details). More positive samples were found with the in sELISA kit than with the other kits we used to evaluate the same samples. To further elucidate this difference, we performed viral isolation and identification, and we found that ALV-K was detected in samples that were positive by sELISA but negative by commercial ELISA. Sequence analysis revealed that the ALV-K virus identified was a recombinant virus with the *env* gene from an exogenous virus and a long terminal repeat (LTR) from an endogenous virus [10]. Although ALV-K can efficiently replicate in DF-1 cells in vitro, ALV-K shows a poor replication ability and reduced viral shedding in infected chickens [11]. Therefore, the level of ALV-K viral shedding in the meconium of infected chickens should be lower than that of other exogenous ALV subtypes, such as ALV-J. Although both ELISAs could detect p27 after passaging the samples in DF-1 cells, the level of p27 in the meconium was low. Thus, more sensitive ELISAs, such as sELISA, are urgently needed.

**Table 2:** Sensitivity for ALV detection compared among different ELISA kits. A. Comparison of the sensitivity of ALV-A detection among different ELISA kits. B. Comparison of the sensitivity of ALV-J detection among different ELISA kits

	1:2^1^	1:2^2^	1:2^3^	1:2^4^	1:2^5^	1:2^6^	1:2^7^	1: 2^8^	1:2^9^	1:2^10^	1:2^11^
A. ALV-A sample dilution											
Commercial ELISA	1.413	1.444	1.345	1.269	0.993	0.695	0.45	0.272	0.11	0.085	0.08
sELISA	1.188	1.207	1.208	1.161	1.148	0.989	0.759	0.511	0.348	0.234	0.17
B. ALV-J sample dilution											
Commercial ELISA	1.563	1.497	1.442	1.354	1.164	0.86	0.607	0.322	0.138	0.098	0.084
sELISA	1.332	1.29	1.312	1.291	1.287	1.253	1.149	0.863	0.656	0.424	0.281

Notably, in 2008 and 2013, the ALV strains PDRC-3249 and TW-3593 were isolated from contaminated commercial Marek’s disease vaccines in the United States and blood samples in Taiwan, respectively [12, 13]. It has been proposed that the PDRC-3249 and TW-3593 strains are possibly the result of recombination between ALV-A and ALV-E viruses. It is interesting that the complete sequences of these two strains are almost the same as those of ALV-K strains. In 2016, Li et al. isolated and identified an ALV-K strain from a local Chinese yellow broiler chicken in southern China [14]. Li’s report showed that the ALV-K strain replicated more slowly and with less pathogenicity than the other ALV subgroup viruses tested. It was hypothesized that the LTR region originating from ALV-E might contribute to the low viral replication, pathogenicity and oncogenesis of ALV-K. This LTR region may be the major reason for the inability to detect the new ALV subgroup with some commercial ELISA kits. In this study, although ALV was not isolated from all 10 samples, the 100% positive rate of the sELISA kit together with the PCR and sequencing results confirmed that all 10 samples were ALV positive. It is suggested that it is challenging to detect the new ALV subgroup with the detection kits that are applied in the current ALV eradication program.

Based on the detection results for the field samples, we showed that the sELISA kit had a higher sensitivity than the commercial kit used in this study. With the application of this sELISA kit, successful detection of ALV-K with a low replication ability is possible, and efficiently apply this sELISA kit to the ALV eradication program will support the control of ALV-K.

## Conclusions

One commercial ELISA kit did not detect all positive samples containing new emerging ALV-K viruses. The new emerging ALV-K strains could be a major challenge to the poultry industry. The higher sensitivity of the sELISA kit used in this study may be very useful in the program for the eradication of ALV.

## Methods

### Chicken farm and samples

All chickens and samples were obtained from a breeding broiler chicken farm in Nanjing, China. An outbreak of ALV-J emerged before 2010 in this area. In this study, 1812 meconium samples were collected from hatched one-day-old chickens on the farm. The samples of cloacal swab, blood and spleen were collected from 10 chickens at 2 days of age, which meconium samples were positive in sELISA kit but negative in commercial ELISA kit. Blood samples were directly taken from the jugular vein. Spleen samples were taken from the chickens, which were first anesthetized by ether inhalation and then sacrificed by exsanguination. Other positive chickens were lately sent the laboratory to be euthanized. All the experiments complied with institutional animal care guidelines and were approved by the University of Yangzhou Animal Care Committee (Authorized XYSK(Su)2016–0020).

### sELISA kit

The sELISA kit applied in China was developed using two mAbs (5D3 and 4F12) against the capsid protein p27 of ALV; this kit was generated and characterized in our previous work [5, 15, 16]. The two mAbs were purified with a protein G column. In the ELISA, the purified mAb 5D3 was used as the coating antibody, and the mAb 4F12 was labeled with HRP and used as the detection antibody. In brief, the purified mAb 5D3 was used to coat 96-well microplates at a concentration of 0.35 μg/mL in 0.1 M carbonate/bicarbonate buffer at pH 9.6 overnight at 4 °C and was then blocked with 5% skim milk in a 10 mM phosphate buffered saline (PBS) solution at pH 7.4 containing 0.05% Tween-20 (PBST) for 2 h at 37 °C. The plate was washed three times with PBST before adding 100 μL of sample. A noninfected DF1 cell supernatant sample and a purified recombinant p27 protein expressed in *Escherichia coli* were used as negative and positive controls, respectively. The plate was incubated for 1 h at 37 °C and washed three times. Then, 100 μL of HRP-labeled 4F12 diluted in PBST was added to the wells, and the plate was incubated at 37 °C for 1 h. After another 5 washes, 100 μL of freshly prepared TMB substrate solution was dispensed into each well. Color development occurred in the dark for 15 min, and the reaction was stopped by adding 100 μL of 1% sodium dodecyl sulfate (SDS). The absorbance at 650 nm was measured using an ELISA reader.

### Commercial ELISA kit

Briefly, samples (blood or tissue) were inoculated into DF-1 cells, which were then incubated at 37 °C in a 5% CO_2_ incubator for 7 days. After the incubation, cell lysates were prepared by 2 freeze-thaw cycles and tested for an ALV group specific antigen (p27) by an antigen capture ELISA using anti-p27 antibody-coated plates (IDEXX Laboratories Pty. Ltd., Westbrook, USA) according to the manufacturer’s protocol. Other samples (meconium) could be tested directly. Samples (100 μL), including positive and negative controls, were added to the ELISA plate wells and incubated at room temperature for 60 min, followed by three to five washes with 350 μL/well washing buffer. Then, 100 μL of an HRP-conjugated rabbit antibody against p27 were added to all the wells, and the plate was incubated for another 60 min. After 3–5 washes, 100 μL of substrate solution was added to develop color for 15 min, and the reaction was stopped by adding 100 μL of stop solution to each well. The absorbance at 650 nm was measured using an ELISA reader.

### Virus isolation

The chicken DF-1 cell line (an immortalized cell line of chicken embryonic fibroblasts, provided by Dr. Lucy Lee, USDA) was used for virus isolation (kept in our laboratory). The cells were grown in Dulbecco’s modified Eagle medium (DMEM) with 5% fetal bovine serum (FBS) and were maintained in DMEM with 1% FBS at 37 °C in a humidified atmosphere containing 5% CO_2_. Before inoculation, DF-1 cells were seeded in 96-well plates and cultured for 24 h. The following day, 60 μL of isolated lymphocytes or tissue homogenate supernatant and 120 μL of DMEM were added and incubated for 2.5 h before replacement with fresh DMEM maintenance medium containing 1% FBS. The plates were then incubated in a culture chamber for 7 days.

### PCR

Viral DNA was prepared using the phenol-chloroform method [17]. The *env* gene of ALV was amplified using DNA extracted from lysed tissue samples as the template with the primers listed in Additional file 1: Table S1. In the PCR system, a 50-μL reaction volume was used, and the reaction mixture consisted of 5 μL of 10× LA Taq PCR buffer, 2 μL of each primer (25 pmol/μL), 0.5 μL of LA Taq DNA polymerase, 4 μL of 10 mM deoxyribonucleotide triphosphate mixture, 36.5 μL of ddH_2_O, and 2 μL of DNA template. The PCR parameters for the *env* gene were 94 °C for 5 min; 30 cycles of 94 °C for 1 min, 55 °C for 1 min, and 72 °C for 2 min; and then 72 °C for 10 min. The PCR products were separated on a 1% DNA gel.

### Sequence analysis of the viral genome

PCR products were sent to the Beijing Genomics Institute for sequencing. Multiple sequence alignment with other ALVs (Additional file 2: Table S2) was carried out using the sequence analysis software Lasergene7 (DNASTAR) and the NCBI BLAST program (http://blast.ncbi.nlm.nih.gov/Blast.cgi). A phylogenetic tree was constructed using the neighbor-joining method (MEGA 5.0).

### Indirect IFA

Cells infected with or without virus were fixed with an acetone:ethanol solution (3:2) for 10 min after 7 days and washed once with PBS. After blocking with 1% BSA in PBS, the fixed cells were incubated with the mouse anti-ALV p27 mAb 5D3 for 45 min at 37 °C. After three washes with PBS, the cells were incubated with a FITC-conjugated goat anti-mouse IgG antibody (Sigma-Aldrich, USA) for another 45 min. After three washes with PBS, the cells were observed under a fluorescence microscope.

## Supplementary information

**Additional file 1: Table S1.** Primers used to amplify the *Env* gene of ALV.

**Additional file 2: Table S2.** Avian leukosis virus strains used for genome comparison with isolated strains.

## Data Availability

The datasets used and/or analyzed in the current study are available from the corresponding author on reasonable request.

## Abbreviations

ALV: Avian leukosis virus
ALV-J: Avian leukosis virus subgroup J
ALV-K: Avian leukosis virus subgroup K
DMEM: Dulbecco’s modified Eagle’s medium
ELISA: Enzyme-linked immunosorbent assay
e*nv*: Envelope gene
FBS: Fetal bovine serum
LTR: Long terminal repeat.
mAb: Monoclonal antibody
PCR: Polymerase chain reaction
sELISA: Sandwich enzyme-linked immunosorbent assay
