# Supplementary material for: Detection of ALV p27 in cloacal swabs and virus isolation medium by sELISA
Source: BMC Vet Res. 2019 Oct 30;15:383. doi: 10.1186/s12917-019-2150-z (PMC6822435; doi:10.1186/s12917-019-2150-z)
Supplement: Supplementary file 1 — Additional file 1: Table S1. Primers used to amplify the Env gene of ALV. [file 12917_2019_2150_MOESM1_ESM.docx]

**Table S1 Primers used to amplify the *Env* gene of ALV**

| Target | Name | Sequence | Length |
| --- | --- | --- | --- |
| Env | Env-F | GAGGTGACTAAGAAAGATGAGGCGAGCC | 2,200 bp |
|  | Env-R | CCATCAACCCAGGTGCACACCAATG |  |
