# Supplementary material for: Detection of ALV p27 in cloacal swabs and virus isolation medium by sELISA
Source: BMC Vet Res. 2019 Oct 30;15:383. doi: 10.1186/s12917-019-2150-z (PMC6822435; doi:10.1186/s12917-019-2150-z)
Supplement: Supplementary file 2 — Additional file 2: Table S2. Avian leukosis virus strains used for genome comparison with isolated strains. [file 12917_2019_2150_MOESM2_ESM.docx]

**Table S2**

| Subgroup | Isolate | Accession no. (year of isolation/country of origin) |
| --- | --- | --- |
| A | DPRE32 | KM434201 |
|  | MAV-1 | L10922 |
|  | MQNCSU | DQ365814 |
|  | RSA | M27980 |
|  | SDAU09E2 | HM452342 |
| B | MAV-2 | L10924 |
|  | Schmidt-Ruppin B | AF052428 |
|  | SDAU09C2h | HM446005 |
| C | ALV-C | AF033808 |
|  | RSV-Prague C | J02342 |
| D | RSV Schmidt-Ruppin D | D10652 |
| E | ALVE-B11 | KC610517 |
|  | Ev-1 | AY013303 |
|  | HPRS-103 | Z46390 |
|  | NX0101 | DQ115805 |
|  | 0661 | AF247566 |
| Suspected new subgroup | SD110503R | KF738251 |
|  | JS11C1 | KF746200 |
|  | Km_5844 | AB670312 |
|  | TW-3593 | HM582658 |
|  | PDRC-3249 | EU070902 |
|  | GDFX0601 | KP686142 |
|  | JS14CZ01  JS14CZ02 | KY490695  KY490695 |
